# Identification of Roll Defect or Damage Based on Rayleigh Waves and Deep Convolutional Neural Network Models

**DOI:** 10.3390/ma19143089

**Published:** 2026-07-17

**Authors:** Biao Xiao, Yue Zhang, Zhiwei Liu, Maoxun Sun

**Affiliations:** 1 Shanghai Institute of Special Equipment Inspection and Technical Research Co., Ltd., Shanghai 200062, China; xiao98140177@aliyun.com; 2 School of Mechanical Engineering, Nantong University, Nantong 226019, China; yuezhang@ntu.edu.cn; 3 School of Electrical Engineering and Automation, Nantong University, Nantong 226019, China; lzw@stmail.ntu.edu.cn; 4 School of Mechanical Engineering, University of Shanghai for Science and Technology, Shanghai 200093, China

**Keywords:** roll damage, Rayleigh wave, power spectrum image, short-time Fourier transform

## Abstract

It is important to detect the damage in the rollers and repair them since the damage to the rollers has a negative impact on the quality of the rolled products. Identifying the types of damage helps determine the repair process and normal production work. Ultrasonic testing technology has the advantages of large detection depth, accurate defect localization, low cost, convenient use, fast speed, and harmlessness to the human body. In order to improve the intelligence of ultrasonic detection for identifying damages in rollers, this article proposes a deep learning classification method of damages based on Rayleigh wave signals and power spectrum images with specific sampling rate, automatic identification of four common types of damages (void, hole, crack, and adhesion) is achieved by establishing end-to-end learning models for one-dimensional (1D) and two-dimensional (2D) data. Firstly, an organic glass inclined block and a clamping device were designed. In the experiment, time-domain signals were received on the right side of the damaged sample, and signal data sets were established for signals with different sampling rates. Then, the power spectrum image data sets were established after a short-time Fourier transform was performed. Next, a damage detection model is established based on a deep learning framework, which includes ResNet, GoogLeNet, DenseNet, and AlexNet with 1D and 2D convolutional channels to extract signal features for classifying damage. Finally, the performances of DenseNet models with different structures and depths are compared based on key indicators such as accuracy and training time. The experiment demonstrates that under high sampling rate conditions, using the power spectrum image of Rayleigh waves as data input yields better results than directly using Rayleigh wave signals. Moreover, for the power spectrum images of 0.5 MS/s Rayleigh waves, using ResNet-18 to establish a deep learning model can achieve high accuracy and shorter training time.

## 1. Introduction

Cold-rolled [1,2] and hot-rolled [3,4] products are widely used in fields such as construction, aerospace, and new energy vehicles. Some damage may occur due to factors such as materials, processes, equipment, workers, and environment. This damage will affect the mechanical properties of cold-rolled and hot-rolled products. Non-destructive testing can detect cracks, inclusions, patches, pits, etc. [1,3] without damaging cold-rolled and hot-rolled products. Currently, the widely used non-destructive testing methods include visual inspection [1,2,3], ultrasonic testing [5], eddy current testing [6], and magnetic flux leakage testing [7]. Non-destructive testing can detect cracks, inclusions, patches, and pits without damaging the products. The quality of rolled products depends on the surface condition of the rollers, and direct non-destructive testing of rollers is more efficient.

Damage such as adhesion, cracks, and inclusions in the rollers seriously affects the quality of the rolled product [8], so it is necessary to detect and repair the damage in a timely manner. At present, the repair techniques for rollers mainly include resurfacing, welding [9], laser cladding [9], continuous casting [10], etc., and there are significant differences in the repair processes for different types of damage. Therefore, accurately identifying the type of damage can help determine the repair process and carry out maintenance work, avoiding the production of a large number of non-conforming products and reducing economic losses [11]. However, visual inspection and eddy current inspection are affected by the visible light penetration ability and skin effect, and are not sensitive to damage on the sub-surface of the rollers [12,13]. Note that ultrasonic waves can characterize internal defects or damage when they pass through the tested samples.

**Figure 1:**
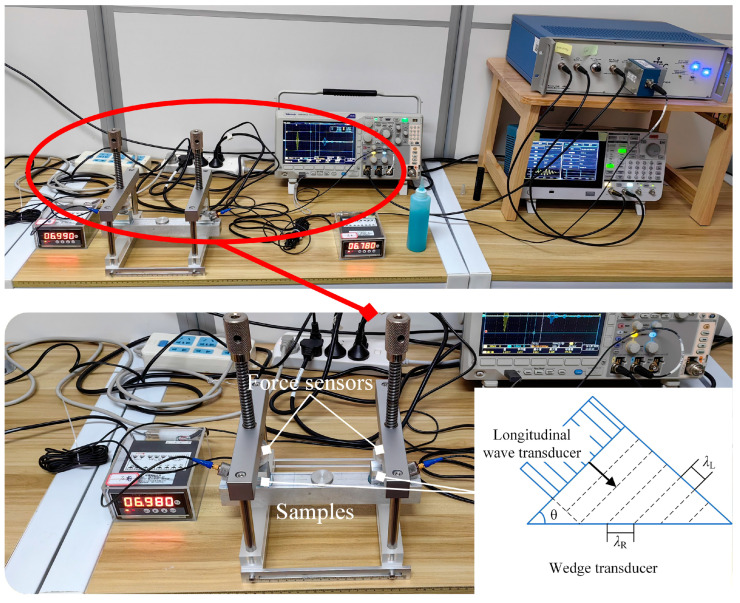
The ultrasonic measurement system.

Ultrasonic testing has become one of the most widely used non-destructive testing techniques [14], and research on material or structural damage detection based on ultrasonic signal processing has developed rapidly. Liu et al. [15] proposed a reconstruction algorithm for probabilistic inspection of defects (RAPID) based on the hierarchical energy distribution for detecting and locating defects in the multi-layer heterogeneous metal-bonded structures (MLHMBSs) that meet the damage detection requirements of long-term service in extreme environments. Due to the combination of the probabilistic damage imaging (PDI) algorithm and a fusion ultrasonic guided wave damage index, which can be used to detect damage in complex composite structures, Jin et al. [16] proposed an improved probabilistic damage imaging algorithm based on the damage shape factor $\beta_{M}$ for identifying multiple defects in curved carbon/epoxy composite structures. The results indicate that this method can effectively identify the number, location, and size of multiple defects present in curved composite structures. Zhang et al. [17] proposed a composite material damage localization imaging method based on the Lamb signal spectrum. Multiple circular sensors are arranged in an array, each rotating clockwise to generate ultrasound waves, while other sensors collect signals, extract the frequency spectrum of the signals to calculate the damage factor, and use probabilistic imaging algorithms to achieve single- and multiple-damage-location imaging. Then, damage imaging localization is achieved through experiments. Compared with bulk waves or ultrasonic Lamb waves, ultrasonic Rayleigh waves are more sensitive to surface and subsurface defects or damages since most of the energy of Rayleigh waves is within the area near the surface. Note that, for the Rayleigh waves, the vector sum of in-plane displacements and out-of-plane displacements gives the ellipse. The elliptical particle motion occurs as a function of depth, with the motion reversing after a particular depth. Most of the energy of Rayleigh waves is within the area near the surface, the depth of which is less than one wavelength. Thus, Rayleigh waves show higher sensitivity to the surface and subsurface defects and damages. As shown in Figure 1, the longitudinal waves can be converted to Rayleigh waves through the Plexiglas wedge.

As a branch and implementation method of artificial intelligence, machine learning learns models based on sample data and uses the models to predict data. Introducing machine learning into ultrasonic non-destructive testing has advantages such as high execution efficiency, strong data execution ability, and strong generalization ability. Fu et al. [18] proposed a composite material damage detection method based on artificial neural networks using laser technology. The energy ratio of signal components was obtained through wavelet packet decomposition, achieving accurate measurement of damage location and size. Oliveira et al. [19] collected ultrasonic signals from turbine blades, performed wavelet denoising on the signals, extracted features based on discrete Fourier transform, and applied principal component analysis. They used k-means, one-class support vector machine (SVM), and distance-based methods to identify the presence of damage, in order to achieve non-destructive ultrasonic testing technology for blades in predictive maintenance. Sun et al. [8] built a measurement system for ultrasonic Rayleigh waves, which receives signals on the left and right sides of roller damage and extracts time-domain features, frequency-domain features, and time-frequency domain features to construct a feature set. By combining classification algorithms such as SVM to build models with swarm intelligence optimization algorithms such as snake optimization (SO) to select features, high classification accuracy has been achieved with fewer features and shorter system consumption time.

Although the introduction of machine learning has greatly assisted in the study of material or structural damage based on ultrasound signals, artificial features rely on specialized knowledge in this application field. To address the issue of poor feature generality, end-to-end deep learning techniques [20] can automatically learn features from large amounts of data and be used for tasks such as classification and localization. Compared with traditional machine learning models, deep convolutional neural network models have better performance and efficiency. In practical applications, researchers continuously improve and innovate convolutional neural network models to enhance detection accuracy and robustness. In damage detection, the design of the network structure is the key to improving detection accuracy. Because the shape, size, location, and other features of the damage are relatively complex, it is necessary to design a targeted network structure to learn these features. Zhang et al. [21] proposed a specific method for damage detection of honeycomb sandwich structures by combining Lamb wave and a one-dimensional convolutional neural network (1D-CNN). Fourier transform was used to convert the signal to the frequency domain and establish a model that can identify the location and degree of internal damage in square honeycomb panels. Cheng et al. [22] used ultrasound to detect the geometric dimensions of internal damage in composite carbon fiber reinforced plastic (CFRP) subjected to low-speed impact and proposed an automatic classification method based on deep learning to estimate the depth of defects. In this method, CNN-LSTM is used to build the model, which achieves more accurate classification of two types of defect samples in CFRP based on a-scan signals compared to CNN or LSTM. Hu et al. [23] combined ultrasound detection with visual localization technology and established an ultrasound signal classification model based on a 1D convolutional neural network to detect internal defects, achieving an interpretation accuracy of 98.74%, thus realizing intelligent interpretation and visual display of internal defects in composite materials. In the research of multi-crack damage identification in aviation structures, Zeng et al. [24] introduced an improved Hausdorff distance-based weight-averaging method for crack location, direction, and quantification, which can detect multi-crack damage through Lamb waves. In addition, deep learning related to ultrasound images is suitable for damage detection of some materials. Kim et al. [25] proposed a damage detection method that combines ultrasound measurements with optical images, extracting ultrasound features from preprocessed ultrasound signals to detect the location of ultrasound emission, and using deep convolutional neural networks to classify imaged objects for damage detection. El Hawwat et al. [26] developed a deep learning model for classifying the severity of cracks based on convolutional neural networks in the study of structural health monitoring of polyethylene pipelines. Continuous wavelet transform was used to convert ultrasound signals into images, achieving accurate crack localization and automatic severity classification.

In this article, an organic glass inclined block and clamping device were designed to maintain the same force between the excitation ultrasonic transducer and the reception ultrasonic transducer in the ultrasonic measurement system. It simulates cavities, voids, cracks, and stuck steel in rollers and receives time-domain signals on the right side of the damage. Based on this, a rolling roll damage classification method based on specific sampling frequency Rayleigh wave signals and power spectrum deep learning is proposed. On the basis of signal segmentation, short-time Fourier transform is used to transform the signal segment into a time-frequency image, and different frequency signal sample datasets are established through downsampling. Based on the 1D convolution method, ResNet, GoogLeNet, DenseNet, and AlexNet are used to construct Rayleigh wave classification models for different damages. Based on the two-dimensional (2D) convolution method, a deep learning network is used to construct image classification models for different types of damage. Model performance is compared based on key indicators such as accuracy and training time. On this basis, further exploration of the structure and parameters of the model is conducted to improve the model performance of the system.

## 2. Experimental Design and Measurements

**Figure 2:**
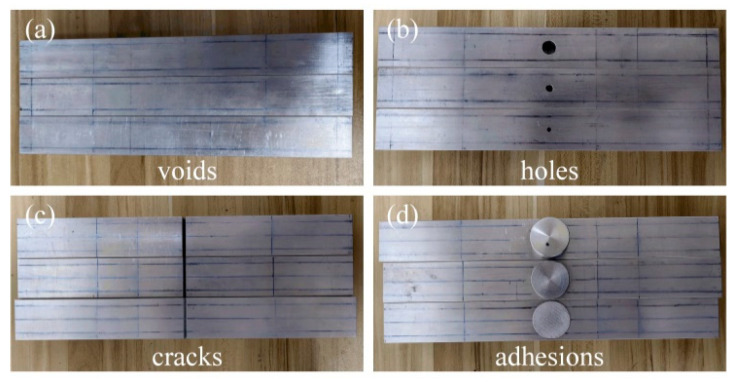
The patterns with different types of damage.

In this study, an ultrasonic measurement system was designed, consisting of an arbitrary function generator, pulse amplifier, oscilloscope, oblique block transducer, clamping device, pressure sensor, etc., as shown in Figure 1. Damaged samples with dimensions of 250 mm, 30 mm, and 30 mm in length, width, and height were prepared to simulate voids, holes, cracks, and adhesion in rollers. Holes with a depth of 27.5 mm and with diameters of 3.1 mm, 5 mm, and 10 mm, respectively, were processed on the pattern. Cracks with a width of 2.5 mm were processed on the sample, with depths of 2.5 mm, 5 mm, and 10 mm, respectively. In addition, the circular disk is adhered to the test sample to simulate adhesion in a roller, with a diameter of 28 mm and thicknesses of 1 mm, 5 mm, and 10 mm, respectively. The pattern with various types of damage is shown in Figure 2. Stimulate ultrasonic Rayleigh waves on the left side of the damage and receive acoustic signals on the right side of the damage.

It is found that, based on our experiments, the time-domain signals at 2.25 MHz or 5 MHz are very weak since higher attenuation may appear with the increase in frequency. In addition, the wavelength of Rayleigh waves at 1 MHz is 2.90 mm, which is smaller than the minimum size of cracks, holes, inclusions, and adhesions. Ultrasonic Rayleigh waves show high sensitivity to damage or defects whose sizes are larger than their wavelength. Therefore, excitation signals are selected as 10-cycle Hanning-windowed sinusoidal tone bursts at 1 MHz. According to formula $\theta =\sin^{-1}(c_{L}^{w}/c_{R}^{s})$, the angle of wedges is selected as 64.5°, where $c_{R}^{s}$ and $c_{L}^{w}$ are the ultrasonic Rayleigh wave velocity (2900 m/s) in the tested sample and ultrasonic longitudinal wave velocity (2617 m/s) in the Plexiglas. Note that $c_{L}^{w}$ must be less than $c_{R}^{s}$. This method is probably the most efficient among normal beam transducer, periodic array, comb transducer, and mediator technique, since Rayleigh waves only propagate in one direction and the calculated value of angle *θ* is independent of the frequencies of Rayleigh waves. The wedge transducer, consisting of a Plexiglas wedge and a narrowband longitudinal wave transducer, is used to generate Rayleigh waves in experiments based on Snell’s law, in which the diameter of the longitudinal wave transducer is 0.71 in. These wedge transducers are coupled to the surface of the samples. To increase the signal-to-noise ratio (SNR), 512 transient responses are measured and averaged in the time domain. It is noted that a specific fixture is designed to ensure the collinear arrangement of transmitters and receivers. Moreover, the contact pressure between the wedge transducers and sample remains constant, making it possible to conduct repeatable measurements and obtain reliable data. Specifically, a fixture and pressure sensor to fix the sample and wedge transducer, with a pressure maintained between 75 N and 80 N, are adopted in the experiment. Rayleigh waves are converted into electrical signals using a wedge transducer and recorded by an oscilloscope with a sampling rate of 5 MS/s. The sizes of the defects or damage are relatively small compared with the diameter of composite rolls (e.g., the radius of the CPC roll in Ref. [9] is 300 mm, while the radius of the hole is roughly 2.5 mm); therefore, we have not taken into account the influence of curvature. In addition, ultrasonic Rayleigh waves can also propagate along the curved surfaces and detect defects or damage.

**Figure 3:**
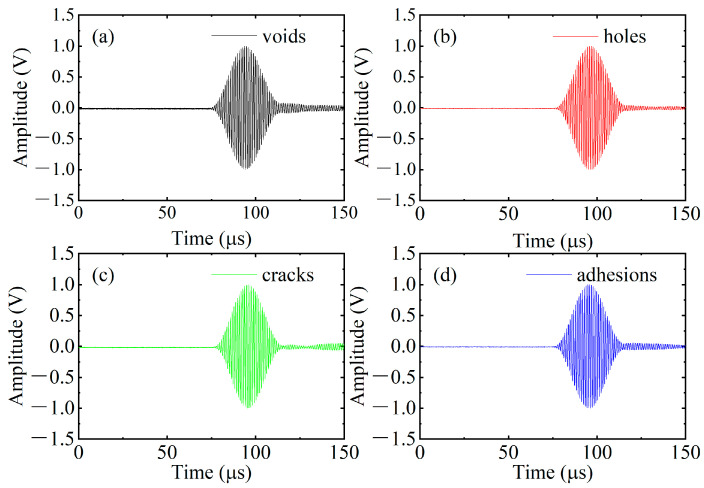
Time domain signals from patterns with (**a**) voids, (**b**) holes, (**c**) cracks, and (**d**) adhesions.

For samples with voids, holes, cracks, and adhesions, the time-domain signal of the ultrasound signal after segmentation and normalization is shown in Figure 3, with significant differences in waveform and requiring further processing. Subsequently, a classification model will be established using deep learning algorithms to automatically extract features and achieve damage recognition through end-to-end methods.

## 3. Methods for Defect or Damage Identification in Composite Rolls

### 3.1. Short-Time Fourier Transformation

For a signal x(t), given a window function with a short-time width that slides over the signal segment, the short-time Fourier transform of the signal is

(1) $$STFT(t,f)=\int_{-\infty }^{+\infty }x(\tau )h(\tau -t)e^{-j2\pi f\tau }d\tau$$

where t is the time, f is the frequency, and $h^{*}(\tau -t)$ represents the complex conjugate function of h(τ−t). For a given time t, consider STFT(t,f) as the power spectrum at that moment. Discretize STFT(t,f) by sampling at the equidistant time-frequency node (mΔt,nΔf), where Δt and Δf represent the sampling intervals of the time and frequency variables, respectively, and the discrete form of s(t) is s(k). The discrete form of the short-time Fourier transform is

(2) $$STFT(m,n)=\sum_{k=-\infty }^{+\infty }s(k)h(k\Delta t-m\Delta t)e^{-j2\pi (n\Delta f)k\left(\Delta t\right)}$$

where h(t) is the window function.

The window function type for the short-time Fourier transform is Hamming, with a window length of nperseg = 320 points. When the overlap ratio is 50%, the frequency resolution is 156.25 kHz, and the calculated time resolution is 6.4 μs. After comprehensive analysis, the STFT parameter optimization strategy is as follows: STFT point number nfft = nperseg (320), no zero padding, frequency point number N/2 + 1 = 161, and standard selection.

### 3.2. Classification Algorithms

Convolutional neural networks were originally designed for image classification tasks, consisting of multiple convolutional layers, each containing multiple convolution kernels. These kernels are used to scan the entire image from left to right and from top to bottom, acquiring the output data called feature maps. After multiple convolution operations, the abstract representation of the image at various scales is finally obtained. Due to its main framework parameters being specific to image data formats, the model is quite large. The more layers a deep learning network has, the stronger its non-linear expression ability and the better the fitting effect. However, simply increasing the depth may lead to gradient vanishing or exploding problems, as well as degradation issues. In this study on damage classification of Rayleigh waves using various convolutional neural network algorithms, multiple convolutional neural networks were designed and applied to ultrasound signal segments and their transformed images.

In this article, 10-fold cross-validation is used for dataset partitioning (Table 1): four types of defect samples are stratified by category and divided into 10 segments each, and combined into 10 subsets; Each iteration takes one subset (10%) as the test set and the remaining nine subsets (90%) as the training set. Divide it into 10 folds (each fold consisting of 1 test set and 9 training sets), and take one fold for each iteration. Divide the training set into 80% for training and 20% for validation. Take turns verifying 10 times to ensure that each subset is evaluated once as a test set, thereby achieving a 9:1 ratio between the training set and the test set.

#### 3.2.1. ResNet

ResNet improves information propagation efficiency by adding directly connected edges to non-linear convolutional layers. In a deep network, use a nonlinear element f(x;θ) to approximate an objective function h(x). However, as the depth of the network increases, degradation occurs, resulting in a decrease in the accuracy of the model on both the training and testing sets. The reason for network degradation is that the multi-layer nonlinear neural network structure is difficult to fit the identity mapping function. Split the objective function into identity function x and residual function h(x)−x [27], let nonlinear unit f(x;θ) approximate residual function h(x)−x, and use f(x;θ)+x to approximate h(x). The residual unit consists of multiple cascaded (cross-layer (equal-width) convolutional layers and a cross-layer (equal-width) convolutional layer and a directly connected edge, which is then activated by ReLU to obtain the output. A residual network is a deep learning network composed of many residual units connected in series. The differences in various ResNet network architecture configurations are reflected in the number of residual layer modules in each layer, as shown in Table 2. During the training process, a back-propagation algorithm based on stochastic gradient descent (SGD) is generally used to optimize model parameters by minimizing the cross-entropy loss function. Data augmentation, regularization, dropout, and other techniques can be used to improve the generalization ability and robustness of the model. By increasing the number of layers in the network, ResNet-18 can be changed to ResNet-34. In addition, for ResNet network architectures with more layers, the Bottleneck structure is adopted, which first reduces the number of channels through convolution. Depending on the number of layers, it can be changed to ResNet-50, ResNet-101, and ResNet-152.

In this article, ResNet achieves implicit regularization through batch normalization (BatchNorm2d). The precise network architecture is ResNet18 (ResNet18), using BasicBlock1 (2-layer residual blocks), with block numbers [2, 2, 2, 2] in each stage. The input dimension is a single-channel grayscale image with a shape of (batch_2, 1, length, channels), batch size of 32, learning rate of 0.001, optimizer of Adam, and training epochs of 80.

#### 3.2.2. DenseNet

DenseNet [28] consists of several dense blocks, which are connected by a transition layer. Each dense block is composed of several convolutional layers, and each convolutional layer has a direct connection path with the previous convolutional layer. The input layer is merged with the outputs of these convolutional layers and used as the input for the current convolution. This preserves all past feature maps, ensuring that the input and output variances of each layer are sufficiently large. The network consists of L layers, each implementing a nonlinear transformation $H_{l}(\cdot )$ and representing the output of the lth layer as $x_{l}$. An additional skip connection is added to bypass the nonlinear transformation through an identity function, which is $x_{l}=H_{l}(x_{l-1})+x_{l-1}$. The identity function combined with the output of $H_{l}$ through summation may hinder the flow of information in the network. In order to further improve the information flow between layers, direct connections between layers and all sublayers are introduced. The lth layer obtains the feature map $x_{l}=H_{l}(\left[x_{0},x_{1},\cdots ,x_{l-1}\right])$ of the previous layer as input, where $\left[x_{0},x_{1},\cdots ,x_{l-1}\right]$ represents the connection that generates the feature map in the 0,⋯,l−1 layers. Define $H_{l}(\cdot )$ as a composite function that includes batch normalization (BN), rectified linear unit (ReLU), and a 3 × 3 convolution. DenseNet is divided into densely connected blocks for downsampling, as the downsampling layer can change the size of the feature map. The layers between dense blocks are called transition layers, which are used for convolution and pooling. In the transition layer, a 2 × 2 pooling layer is set after a normalization layer and a 1 × 1 convolutional layer. When each function $H_{l}$ generates k feature layers, the lth layer has a $k_{0}+k\times (l-1)$ input mapping, where $k_{0}$ is the number of channels in the input layer, and hyper-parameter k is adopted as the growth rate of the network.

DenseNet achieves implicit regularization through batch normalization (BatchNorm2d). The precise network architecture is growth_rate = 16, block_config = (6, 24, 12, 6), initial feature count is 32, input dimension is a single-channel grayscale image, shape is (batch_2, 1, length, channels), batch size is 32, learning rate is 0.001, optimizer is Adam, and training rounds are 80.

#### 3.2.3. AlexNet

AlexNet network [29] has 5 convolutional layers and 3 fully connected layers, of which 3 convolutional layers are connected to the max-pooling layer, and the last layer is the SoftMax output layer, with the number of nodes corresponding to the number of classifications. The dropout operation mechanism is adopted in AlexNet, and a portion of neurons are randomly selected for forward and backward propagation during training, while keeping the parameter values of other neurons unchanged to alleviate overfitting. AlexNet is learned through the kernels in each convolutional layer, and each convolution generates a feature map. Sampling is done in the mapping layer, and the kernel is convolved with the feature maps in the previous layer and combined with weights to generate the feature maps in the next layer. Each feature map is followed by an activation function to introduce nonlinear factors to improve the classification performance of the data. In order to achieve translation invariance of the input image and reduce the weight of each layer by shrinking the image, the feature mapping is resampled using max-pooling, which captures the maximum value of the feature map in the neighborhood. According to the design of local connections and shared weights, the learning mode between the input layer and the output layer may vary.

Two layers of Dropout are continuously used in the AlexNet fully connected layer (fc), with a probability of 0.5 for both. The precise network architecture uses AdaptiveAvgPool2d to input a single-channel grayscale image with a shape of (batch_2, 1, length, channels), a batch size of 32, a learning rate of 0.001, an optimizer of Adam, and 80 training rounds.

#### 3.2.4. GoogLeNet

Inception mechanism is adopted in GoogLeNet [30] and multi-scale is applied to process images. Its advantage is to significantly reduce the number of model parameters, integrate multiple convolution kernels and pooling of different scales, and form an Inception module. This module consists of three sets of convolutional kernels and a pooling unit, which jointly accept input images from the previous layer. There are three sizes of convolutional kernels and a max-pooling operation, which process the input images in parallel and then concatenate the input results according to channels. Because the convolution operation accepts input images of equal size, and the convolution performs a padding operation, the size of the input images is also the same, and can be directly concatenated according to channels. To ensure that the size remains unchanged after the pooling operation, pooling with a step size of 1 is adopted.

Dropout (0.5) is used in the fully connected layer of the GoogLeNet-assisted classifier. The precise network architecture includes two auxiliary classifiers, InceptionAux. The input dimension is a single-channel grayscale image with a shape of (batch_2, 1, length, channels), batch size is 32, learning rate is 0.001, optimizer is Adam, and training rounds are 80.

### 3.3. Evaluation Method

Suspendisse In order to further evaluate the performance of the model established by combining deep learning methods with signals and images as inputs, a portion of the samples are selected as the training set and another portion as the testing set. Evaluation metrics are calculated based on the output results of the model on the testing set. In order to further evaluate the performance of the model established by combining deep learning methods with signals and images as inputs, a portion of the samples are selected as the training set and another portion as the testing set. Evaluation metrics are calculated based on the output results of the model on the testing set. Cross-validation randomly and uniformly divides the sample into K parts, with K−1 parts used to train the model at a time, and the remaining 1 part used as the test set for the model. The mean of the indicators obtained from K test sets is used, and 10-fold cross-validation is used here.

Using TP to represent the true example, that is, the number of samples correctly classified as positive categories by the model; TN represents true negative examples, which refers to the number of samples correctly classified as negative by the model; Using FP to represent false positives, the number of samples misclassified as positive by the model; Using FN to represent false negative examples, the number of samples misclassified as negative by the model. The formula for calculating accuracy is $accuracy=\frac{TP+TN}{TP+TN+FP+FN}$. In this article, the accuracy of multi-classification problems (void, hole, crack, and adhesion) is evaluated using confusion matrices. In this article, the accuracy of multi-classification problems (void, hole, crack, and adhesion) is evaluated using a confusion matrix. For N-class classification problems, the element $c_{ij}$ of the confusion matrix (N × N) represents the proportion of ith class samples judged by the classifier as jth class.

To measure the performance of the model, precision, recall, and *F*1 value are used as evaluation indicators for the model’s effectiveness. The formula is as follows:

(3) $$precision=\frac{TP}{TP+FP}$$

(4) $$recall=\frac{TP}{TP+FN}$$

(5) $$F1=2\times \frac{precision\times recall}{precision+recall}$$

where *TP* is the number of true examples, *TN* is the number of true negative examples, *FP* is the number of false positive examples, and *FN* is the number of false negative examples. The horizontal axis of the curve represents the false positive rate $\mathrm{FPR}=\frac{FP}{FP+TN}$, and the vertical axis represents the true positive rate $\mathrm{TPR}=\frac{\mathrm{TP}}{\mathrm{TP}+\mathrm{FN}}$. The *AUC* value is the area under the *ROC* curve, with a range of values between [0, 1]. More details can be seen in Table 3.

## 4. Results and Discussions

In this section, the Rayleigh wave signals with different types of damage are first downsampled and segmented, and the signal segment samples with different sampling rates are converted into power spectrograms through short-time Fourier transform. Next, multiple different convolutional neural network algorithms were used to establish deep learning models for one-dimensional signals and two-dimensional images, and the impact of sampling rate, data type, and algorithm on the overall classification accuracy and various recognition rates of the system was analyzed. Finally, further research was conducted on convolutional neural networks with better classification results using different structures and parameters, in order to achieve high classification accuracy and good performance with short training time.

### 4.1. Down Sampling and Imaging of Rayleigh Waves

**Figure 4:**
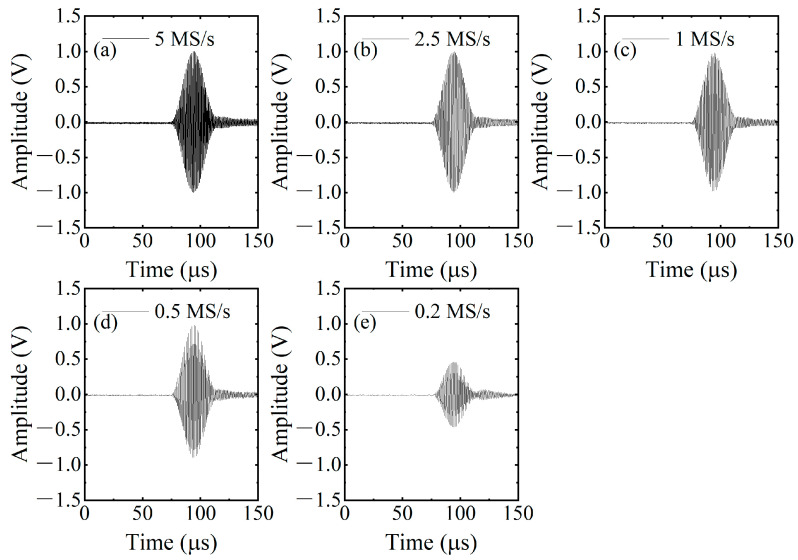
Rayleigh waves with different sampling rates: (**a**) 5 MS/s; (**b**) 2.5 MS/s; (**c**) 1 MS/s; (**d**) 0.5 MS/s and (**e**) 0.2 MS/s.

As the industrial signals contain more valuable information in certain specific frequency bands [18], the sampling rate of Rayleigh waves may affect the training time and effectiveness of damage classification models. According to the Nyquist criterion, in frequency domain analysis, when the sampling rate is greater than twice the highest frequency of the excitation signal, the collected data points can contain all frequency domain information of the excitation signal. In the experiment of this study, the excitation signal was set to 1 MHz, and when using a sampling rate of 2 MS/s or higher, the effective frequency domain information will be completely present in the sampled signal segment. Using a high sampling rate means that it is possible for more effective but unknown time-domain features to be utilized by deep learning models, which can have a positive impact on the final classification accuracy. As the signal sampling rate decreases, on the one hand, the burden of signal processing and model training on the system is reduced to improve operational efficiency; on the other hand, when the sampling rate decreases to a certain extent, useful information will be lost, which will have a negative impact on classification accuracy. Therefore, it is necessary to explore the relationship between ultrasound signal sampling rate and deep learning modeling performance. Starting from a sampling rate of 5 MS/s, the signal is downsampled to 2.5 MS/s, 1 MS/s, 0.5 MS/s, and 0.2 MS/s. After segmentation, a signal segment is obtained, and the signal segments obtained after downsampling at different ratios are shown in Figure 4. Here, when the sampling rate of the signal segment is reduced to 0.5 MS/s, the waveform of the signal remains similar to the original signal segment (5 MS/s); When the sampling rate of the signal segment is reduced to 0.2 MS/s, the waveform of the signal segment shows significant distortion.

**Figure 5:**
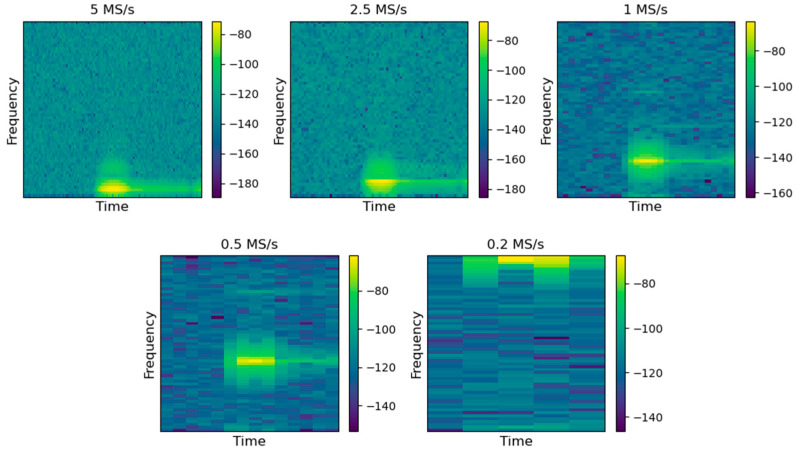
Rayleigh power spectrum images based on short-time Fourier transformation.

The power spectrum image of Rayleigh waves is obtained by using the short-time Fourier transform [31] for each signal segment. The power spectrum images of different sampling rates are shown in Figure 5, where the horizontal axis is time and the vertical axis is frequency. The image represents the power spectral density of the signal at different frequencies at each time, in decibels (dB). Here, the color bar is only used to express numerical values and is not used for model training. When the sampling rate of the signal segment decreases to 0.2 MS/s, the maximum amplitude value appears at the boundary. The signal segment and the power spectrum image obtained by short-time Fourier transform are taken as samples, and various deep convolutional neural network frameworks are used to train the classification model. Then, the best modeling method is selected by evaluating the classification results and comparing the training time.

### 4.2. Comparison of Deep Convolutional Neural Networks for Ultrasound Signal Input

**Figure 6:**
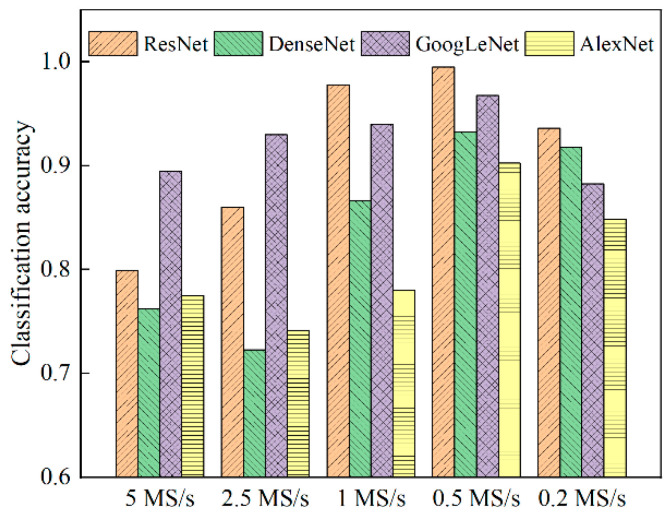
Damage classification accuracy based on one-dimensional Rayleigh signals using convolutional neural networks.

**Figure 7:**
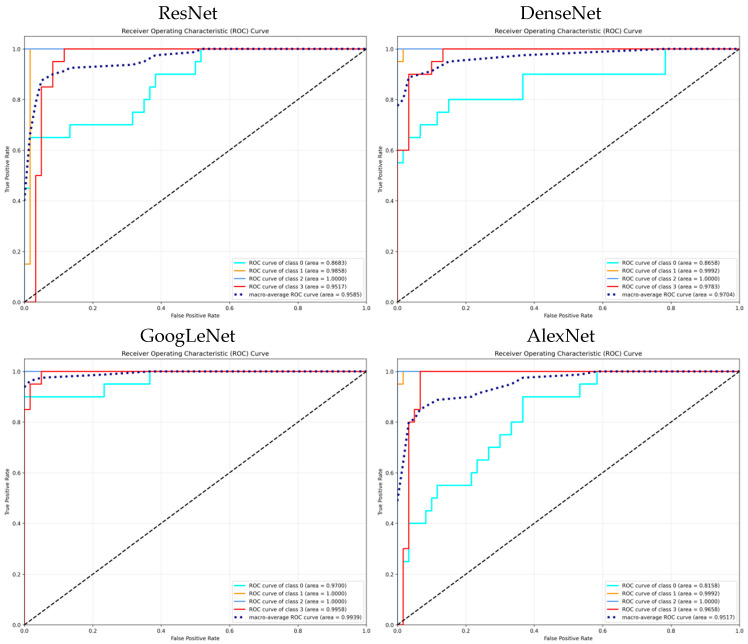
ROC curves of four models.

**Figure 8:**
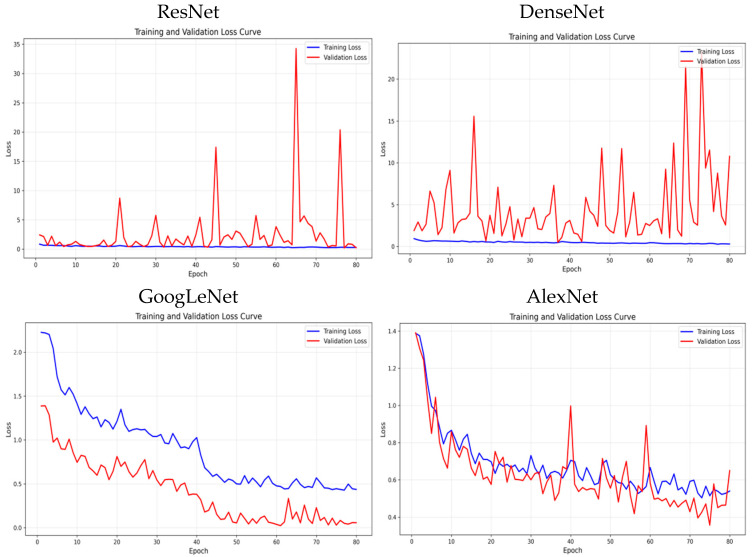
Model training and validation accuracy/loss variation curve.

On a dataset consisting of one-dimensional ultrasound signal samples with different sampling frequencies, deep convolutional neural network frameworks such as ResNet-18 were used for training. The classification results of 10-fold cross-validation are shown in Figure 6. At present, various Convolutional Neural Networks (CNNs) have been used in engineering signal deep learning research in some fields [32,33,34]. In this study, when the sampling rates were 5 MS/s and 2.5 MS/s, GoogLeNet was used to train the deep learning model, resulting in higher classification accuracy. However, when the sampling rate is below 2.5 MS/s, the classification accuracy obtained using ResNet-18 is significantly higher than that of the other three models, especially reaching 0.995 (with a sampling rate of 5 MS/s). In addition, for the other three algorithms, the classification accuracy of the model trained on the 5 MS/s signal dataset is higher than that of other signal datasets with different sampling rates. The average training time of the model is shown in Table 4, and the results indicate that as the sampling rate decreases, the average training time decreases significantly. When the sampling rate is between 5 MS/s and 0.5 MS/s, training a deep learning model using ResNet-18 takes less time than using the other three convolutional neural network frameworks. ROC curves, model training, and validation accuracy/loss variation curves for the four models can be seen in Figure 7 and Figure 8.

**Figure 9:**
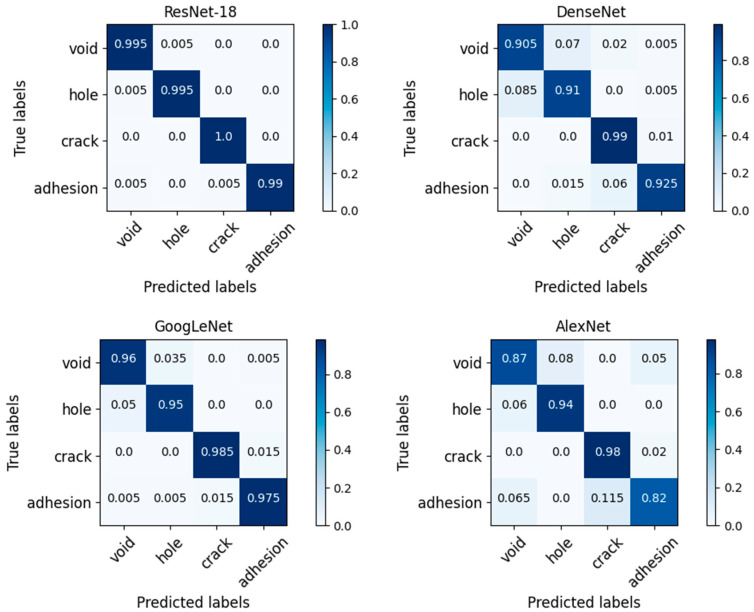
Confusion matrix obtained by training models using different convolutional neural networks based on 1D signals.

When the signal sampling rate is 5 MS/s, four convolutional neural network frameworks are used to train the deep model and obtain the confusion matrix as shown in Figure 9. When using ResNet-18, the recognition rate of crack is 1, and there are a few recognition errors in the other three categories. When using other convolutional neural network frameworks, there is a certain degree of recognition error for each category of damage, especially for AlexNet, which has a low recognition rate of only 0.87 for void.

### 4.3. Comparison of Deep Convolutional Neural Networks for Power Spectrum Image Input

**Figure 10:**
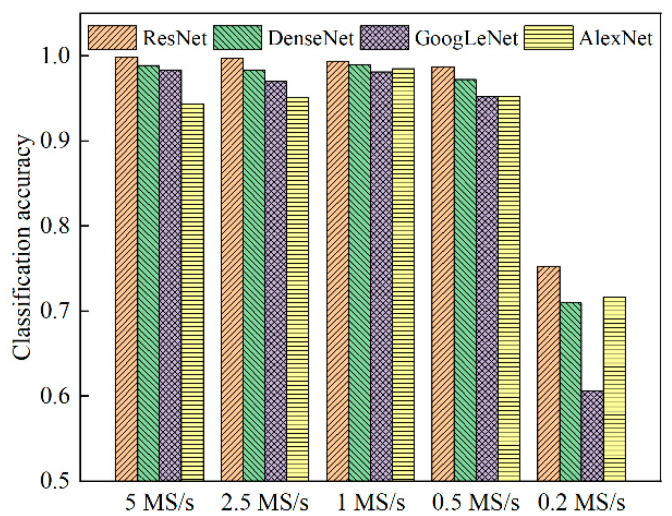
Damage classification accuracy based on short-time Fourier images using convolutional neural networks.

For Rayleigh signals with different sampling rates, the short-time Fourier transform is used to obtain power spectrum images and establish a dataset. The classification models are trained using the four convolutional neural network frameworks mentioned above. The classification results of 10-fold cross-validation are shown in Figure 10. When the sampling rate is between 50 M and 5 M, the classification accuracy of models trained using ResNet-18 and DenseNet is relatively high. Especially when the sampling rate is 5 MS/s or 2.5 MS/s, the classification accuracy obtained using ResNet-18 reaches the highest, which is 0.998. When the sampling rate of the signal is MS/s, the classification accuracy of the model significantly decreases, and even with ResNet-18, the classification accuracy obtained is only 0.75.

The average training time of the model is shown in Table 5, and the results indicate that as the sampling rate decreases, the average training time decreases significantly. When the sampling rate is between 5 MS/s and 0.5 MS/s, training a deep learning model using ResNet-18 takes less time than using the other three convolutional neural network frameworks.

**Figure 11:**
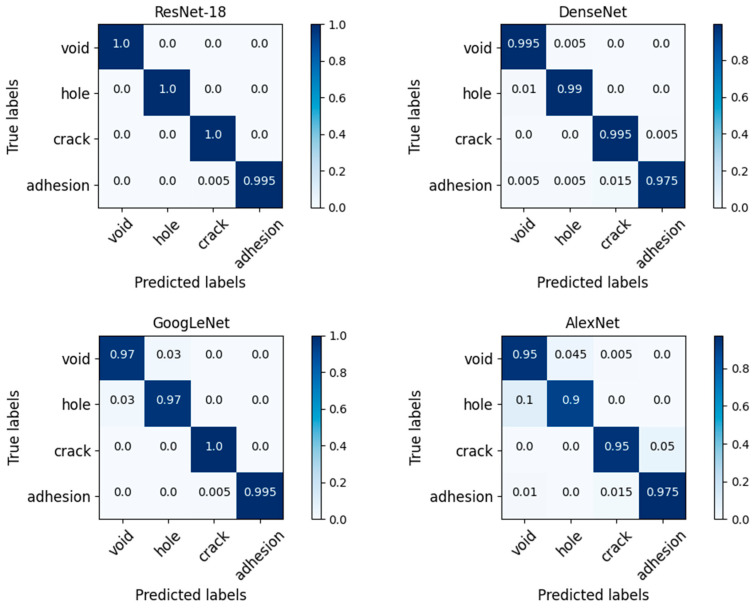
Confusion matrix obtained by training models with different convolutional neural networks based on 2D power spectrum images.

When the signal sampling rate is 5 MS/s, four convolutional neural network frameworks are used to train the deep model and obtain the confusion matrix as shown in Figure 11. When using ResNet-18, the recognition rates for crack, hole, and crack are all 1. When using other convolutional neural network frameworks, there is a certain degree of recognition error for each category of damage, especially for AlexNet, which has a low recognition rate of only 0.87 for void.

ResNet-18 effectively alleviates the problem of gradient vanishing in deep networks through residual connections, allowing the temporal features of the Rayleigh wave to be directly transmitted to the decision layer. The moderate depth of its 18 layers precisely matches the size of the Rayleigh wave data, avoiding overfitting. Compared with DenseNet’s feature redundancy accumulation, GoogLeNet’s multi-branch complex structure, and AlexNet’s insufficient shallow feature extraction, ResNet-18 achieves optimal extraction and fusion of multi-scale features of Rayleigh waves with a concise and efficient architecture, thus achieving the best classification performance among the four CNN models.

### 4.4. Comparison of Classification Results of ResNets with Different Structures

**Figure 12:**
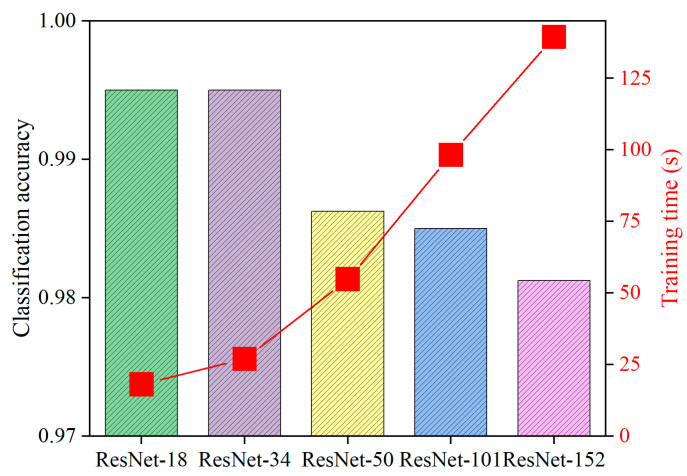
Classification accuracy and training time (red square) of the ResNet model based on the 1D Rayleigh signal.

Due to the fact that the classification accuracy obtained by training the model using ResNet-18 is the highest when the sampling rate of the 1D ultrasound signal is 0.5 MS/s, it is considered to change the number of layers and structure of ResNet, that is, to establish classification models for the 0.5 MS/s ultrasound signal dataset using ResNet-34, ResNet-50, ResNet-101, and ResNet-152, respectively. The classification accuracy of the damage and the training time of the model are shown in Figure 12 to evaluate the performance of the deep learning network in learning the 1D signal dataset. As the model hierarchy deepens, the average classification accuracy decreases from 0.995 to 0.981, and the average training time increases significantly from 18.05 s to 139.44 s. Therefore, with the increase in model layers, although the fitting ability is enhanced, the performance of the model in the classification problem of the Rayleigh wave signal dataset shows a significant decline.

**Figure 13:**
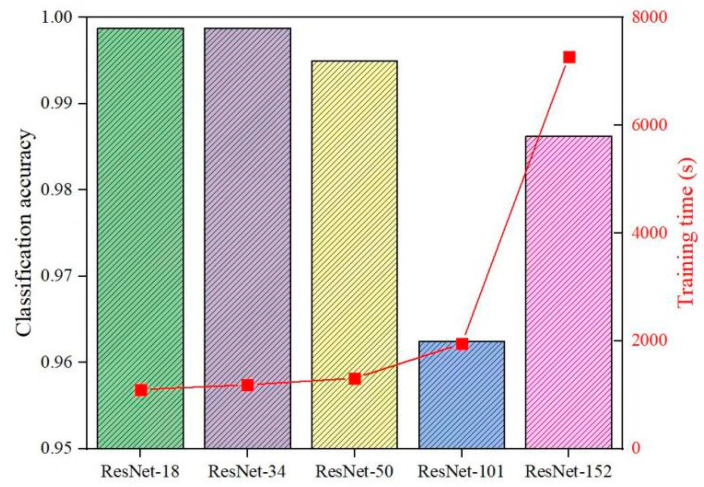
Classification accuracy and training time (red square) of the ResNet model based on the power spectrum of Trum images.

ResNet-34, ResNet-50, ResNet-101, and ResNet-152 were used to establish classification models for 5 MS/s power spectral image datasets, and the classification accuracy of damage and the training time of the models are shown in Figure 13. The performance of deep learning networks in learning 2D image datasets was evaluated, and the performance of deep learning networks in learning 2D image datasets was evaluated. Compared with 1D signals, using 2D image data as input increases the training time of the model by an order of magnitude, resulting in better classification results. As the model hierarchy deepens, the average classification accuracy decreases from 0.998 to 0.987, and the average training time increases significantly from 1101.14 s to 7273.45 s. Therefore, with the increase in model layers, although the fitting ability is enhanced, the performance of the model in the classification problem of the Rayleigh wave image dataset also shows a significant decline.

The computational speed of one-dimensional signal input is much faster than that of two-dimensional power spectrum image input. In terms of memory consumption, the four models for one-dimensional input memory consumption are ResNet with a parameter size of about 11.2 M and a memory size of about 45 MB, DenseNet with a parameter size of about 5.0 M and a memory size of about 20 MB, GoogLeNet with a parameter size of about 6.8 M and a memory size of about 27 MB, AlexNet with a parameter size of about 2.5 M and a memory size of about 10 MB, ResNet with a parameter size of about 60.2 M and a memory size of about 241 MB, DenseNet with a parameter size of about 8.0 M and a memory size of about 32 MB, respectively. The parameter size of GoogLeNet is about 6.8 M, and the memory is about 27 MB, while the parameter size of AlexNet is about 62.4 M and the memory is about 250 MB.

The following conclusion can be drawn from the comparison of computational complexity: The input shape of a one-dimensional input is (N, 1, L, 1), the convolution kernel is (k, 1) one-dimensional, and the feature map dimension only changes along the length direction, resulting in relatively low computational complexity. The input shape of the two-dimensional input is (N, 3, 224, 224), the convolution kernel is (k, k) two-dimensional, and the feature map dimension varies bidirectionally along height × width, resulting in relatively high computational complexity.

The inference speed of one-dimensional signal input is significantly faster than that of two-dimensional power spectrum image input. The core reason is that one-dimensional convolution kernels (such as 3 × 1) only operate on a single dimension, and the effective dimension of feature maps is much lower than the H × W bidirectional expansion of two-dimensional convolution, which reduces the overall floating-point operation times by 10–100 times; At the same time, one-dimensional data itself has minimal memory usage, lower data I/O and preprocessing overhead, and higher cache hit rate.

### 4.5. Scientific Findings, Limitations, and Future Research

In the past, non-destructive testing studies based on ultrasound signals directly processed 1D signals into time-frequency images [34]. There have also been studies on deep learning modeling based on ultrasound signals to time-frequency images, and satisfactory results have been achieved [32]. The main benefit of converting signals into spectrograms for analysis is to intuitively reveal the energy distribution of signals at different frequencies, making it easier to identify dominant frequencies. According to the research results of this article, the recognition results obtained by training the classification model using short-time Fourier images are more stable when the sampling rate decreases, while the recognition results obtained by directly training the classification model using 1D signals fluctuate greatly when the sampling rate decreases. Increasing the sampling rate at the same time has no further effect on the accuracy of the model based on 2D spectrogram images, but the accuracy of the model based on the time-domain signal will have uncertain changes.

This study provides an intelligent identification method based on ultrasonic non-destructive testing for discovering damage to rolling rolls, laying the foundation for the development of future testing equipment. More results about the stable performance of deep learning models should be demonstrated in practical field applications.

In future research, we plan to conduct further analysis on the damage of specimens made of specific materials, considering increasing the types of damage and using different degrees for each type of damage; expand the sample size and dataset of ultrasound signals, conduct more research on deep learning modeling methods for damage identification; and further explore intelligent quantitative analysis of material damage classification and degree based on ultrasound non-destructive testing technology.

## 5. Conclusions

In this article, a roller damage classification method based on specific sampling rate Rayleigh wave signals and power spectrum images using deep learning is proposed for automatic identification of four common types of roller damage, namely voids, holes, cracks, and adhesions. Using 1D signal data as input, the classification accuracy shows an initial increase followed by a decrease as the sampling frequency decreases. The classification accuracy obtained using a sampling frequency of 0.5 MS/s is the highest. Using 2D image data as input, the classification accuracy obtained is relatively high when the sampling frequency is 5 or 2.5 MS/s, and there is little change in the process of reducing to 0.5 M times. For different convolutional neural network algorithms, using ResNet-18 to establish a deep learning model yields better classification results than using other algorithms. The final research results indicate that using ResNet-18 to establish a deep learning model for 0.5 MS/s sampled Rayleigh wave power spectrum images as data input can achieve a classification accuracy of up to 0.98.

## Figures and Tables

**Table 1:** The information about the 10-fold cross-validation of the samples.

Defect Type	Number of Samples	Training	Validation	Testing
Void	200	144	36	20
Hole	200	144	36	20
Crack	200	144	36	20
Adhesion	200	144	36	20

**Table 2:** Residual layer parameters in various ResNet network architecture configurations.

	ResNet-18	ResNet-34	ResNet-50	ResNet-101	ResNet-152
residual layer 1	maximum pooling layer, 3 × 3, stride = 2				
	$\left[\begin{array}{cc} 3\times 3, & 64\\ 3\times 3, & 64 \end{array}\right]\times 2$	$\left[\begin{array}{cc} 3\times 3, & 64\\ 3\times 3, & 64 \end{array}\right]\times 3$	$\left[\begin{array}{cc} 1\times 1, & 64\\ 3\times 3, & 64\\ 1\times 1, & 256 \end{array}\right]\times 3$	$\left[\begin{array}{cc} 1\times 1, & 64\\ 3\times 3, & 64\\ 1\times 1, & 256 \end{array}\right]\times 3$	$\left[\begin{array}{cc} 1\times 1, & 64\\ 3\times 3, & 64\\ 1\times 1, & 256 \end{array}\right]\times 3$
residual layer 2	$\left[\begin{array}{cc} 3\times 3, & 128\\ 3\times 3, & 128 \end{array}\right]\times 2$	$\left[\begin{array}{cc} 3\times 3, & 128\\ 3\times 3, & 128 \end{array}\right]\times 3$	$\left[\begin{array}{cc} 1\times 1, & 128\\ 3\times 3, & 128\\ 1\times 1, & 512 \end{array}\right]\times 4$	$\left[\begin{array}{cc} 1\times 1, & 128\\ 3\times 3, & 128\\ 1\times 1, & 512 \end{array}\right]\times 4$	$\left[\begin{array}{cc} 1\times 1, & 128\\ 3\times 3, & 128\\ 1\times 1, & 512 \end{array}\right]\times 8$
residual layer 3	$\left[\begin{array}{cc} 3\times 3, & 256\\ 3\times 3, & 256 \end{array}\right]\times 2$	$\left[\begin{array}{cc} 3\times 3, & 256\\ 3\times 3, & 256 \end{array}\right]\times 6$	$\left[\begin{array}{cc} 1\times 1, & 256\\ 3\times 3, & 256\\ 1\times 1, & 1024 \end{array}\right]\times 6$	$\left[\begin{array}{cc} 1\times 1, & 256\\ 3\times 3, & 256\\ 1\times 1, & 1024 \end{array}\right]\times 23$	$\left[\begin{array}{cc} 1\times 1, & 256\\ 3\times 3, & 256\\ 1\times 1, & 1024 \end{array}\right]\times 36$
residual layer 4	$\left[\begin{array}{cc} 3\times 3, & 512\\ 3\times 3, & 512 \end{array}\right]\times 2$	$\left[\begin{array}{cc} 3\times 3, & 512\\ 3\times 3, & 512 \end{array}\right]\times 3$	$\left[\begin{array}{cc} 1\times 1, & 512\\ 3\times 3, & 512\\ 1\times 1, & 2048 \end{array}\right]\times 3$	$\left[\begin{array}{cc} 1\times 1, & 512\\ 3\times 3, & 512\\ 1\times 1, & 2048 \end{array}\right]\times 3$	$\left[\begin{array}{cc} 1\times 1, & 512\\ 3\times 3, & 512\\ 1\times 1, & 2048 \end{array}\right]\times 3$

**Table 3:** Additional indicators for model evaluation.

Modeling	*Precision*	*Recall*	*F*1	*AUC*
ResNet	0.9048	0.8875	0.8850	0.9515
DenseNet	0.8333	0.7	0.6760	0.9608
GoogLeNet	0.9286	0.9	0.9010	0.9915
AlexNet	0.8137	0.7875	0.7821	0.9452

**Table 4:** Average training time (s) for classification models of 1D Rayleigh signals with different sampling frequencies.

	5 MS/s	2.5 MS/s	1 MS/s	0.5 MS/s	0.2 MS/s
ResNet-18	89.63	42.95	20.02	14.99	13.57
DenseNet	224.13	94.27	40.52	36.01	36.31
GoogLeNet	171.05	86.69	37.58	27.35	26.22
AlexNet	185.87	92.81	38.08	18.05	7.96

**Table 5:** Average training time (s) for classification models of short-time Fourier transform power spectra with different sampling rates.

	5 MS/s	2.5 MS/s	1 MS/s	0.5 MS/s	0.2 MS/s
ResNet-18	1101.14	1079.12	1086.43	1115.39	1118.89
DenseNet	1192.34	1186.16	1188.13	1206.38	1210.85
GoogLeNet	991.87	974.18	976.58	1002.91	1009.34
AlexNet	1114.75	1104.29	1105.61	1129.74	1131.24

## Data Availability

The original contributions presented in this study are included in the article. Further inquiries can be directed to the corresponding author.
